# “Obesity Paradox” in Acute Respiratory Distress Syndrome: Asystematic Review and Meta-Analysis

**DOI:** 10.1371/journal.pone.0163677

**Published:** 2016-09-29

**Authors:** Guo Zhi, Wang Xin, Wang Ying, Xing Guohong, Liu Shuying

**Affiliations:** Department of Pulmonary Diseases,Jinan Military General Hospital,Jinan, 250031, China; University of Illinois at Chicago College of Medicine, UNITED STATES

## Abstract

**Background:**

It is unclear whether an “obesity paradox” exists in the respiratory system, especially in acute respiratory distress syndrome (ARDS) and acute lung injury (ALI). Previous studies have postulated a causal relation between obesity and ARDS/ALI but have lacked power to form a definitive conclusion.

**Objective:**

To investigate the relationships between obesity, ARDS/ALIrisk, and mortality.

**Methods:**

A systematic search current to April 2016 was performed in Pubmed, EMBASE, Medline, Cochrane databases to find relevant studies. All studies that estimate the effect of obesity in the morbidity and mortality of ARDS/ALI were included.

**Results:**

A total of 24 studies including 9,187,248 subjects were analyzed. The combined results from 16 studies that examined the effect of obesity in morbidity of ARDS/ALI showed an89% increase in odds ratio(pooled odds ratios (OR) 1.89, 95% confidence intervals (CI) 1.45 to 2.47). In subgroup analysis, compared to normal weight, obesity was associated with an increased risk of ARDS/ALI (OR1.57, 95% CI 1.30–1.90 for obese (BMI30-39.9kg/m^2^); OR1.75, 95% CI 1.42–2.15 for obese(BMI≥30kg/m^2^); OR1.67, 95% CI 1.04–2.68 for morbid obese(BMI≥40kg/m^2^)). The combined results from 9 studies that examined the effect of obesity in mortality of ARDS/ALI had a pooled odds ratio(pooled OR 0.63, 95% CI 0.41 to 0.98). Inversely, obesity was significantly associated with reduced risk of ARDS/ALI mortality(OR0.88, 95% CI 0.78–1.00 for overweight(BMI≤18.5m^2^); OR0.74, 95% CI 0.64–0.84 for obese (BMI30-39.9kg/m^2^);OR0.84, 95% CI 0.75–0.94 for 60days mortality; OR0.38, 95% CI 0.22–0.66 for 90days mortality).

**Conclusions:**

Our data identify obesity as an important risk factor for the development of ARDS/ALI; however, ARDS/ALI outcomes are improved in this population when compared to individuals with a normal body mass index. This meta-analysis results supported ‘‘obesity paradox” in ARDS/ALI.

## Introduction

Acute respiratory distress syndrome(ARDS) /Acute lung injury (ALI) is a severe formof acute edematous lung injury which is characterized by arapid development of acute respiratory failure. It develops in response toother serious medical conditions including sepsis, pneumonia, aspiration and trauma[1, 2],and is associated with high morbidity and mortality. Obesity has risen to epidemic proportions in the US and other developed countries[3]. Over one third of the American population is obese, and over 5% is extremely obese[4].Obesity is a risk factor for many diseases including diabetes, dyslipidemia, heart disease, hypertension, insulin resistance, stroke, sleep apnea and cancer[5].However, an inverse relationship between obesity and mortality has been described in patients with cardiovascular disease and diabetes [6–7].This phenomenon is known as the “obesity paradox”.

Some studies of obesity and ARDS/ALI have focused on morbidity with inconsistent results[8–11]. Some studies found obesity to be associated with decreased mortality[12,13], while other studies showed no to improved mortality in the obese patients[8,14].Whether the “obesity paradox” exists in ARDS/ALI is still unclear. With the rising incidence of obesity and the increased mortality, morbidity and health expenditures associated with ARDS/ALI, we present here a systematic review and meta-analys is to investigate the relationships between obesity, ARDS/ALI risk, and mortality.

## Methods

### Data Sources and Search Strategy

We searched several databases (Pubmed, EMBASE, Medline and the Cochrane databases) for relevant studies published through April 2016. ‘‘obesity”, ‘‘body mass index” or “BMI”, combined with ‘‘acute respiratory distress syndrome”, “ARDS”, “acute lung injury” or “ALI” were keywords used for searching. There was no language restriction. Moreover, review and reference lists of relevant articles were screened for additional articles. The searches were conducted independently by two authors (Guo Zhi and Wang Xin).

### Inclusion and Exclusion Criteria

Studies were included if they met the following criteria: (1) reference to human beings;(2)the primary objective was to investigate the effect of BMIon the morbidity and mortality of ARDS/ALI; (3) obesity was categorized by BMI, and BMI was calculated by dividing the patient’s body weight in kilograms by the square of their height in meters.(4)reference to odds ratio (OR), relative risk (RR) or equivalent values representing risks of morbidity and mortality in ARDS/ALI. Studies were excluded if one of the following existed: (1) case reports, editorials, reviews and abstracts; (2) insufficient data to extract or calculate the pooled results. When pertinent data were not included in a published article, the corresponding author was contacted. If a response was not provided, the article was excluded. All the studies were screened independently by two authors (Guo Zhi and Wang Ying). Disagreements were resolved by a third opinion (Liu Shuying).

### Data Extraction and Synthesis

The information of studies included first author, year of publication, study design, sample size, BMI classification,ARDS/ALI diagnostic standard and outcomes. If studies did not report OR, RRand 95% confidence intervals(95%CI), raw data were screened to determine whether ORs could be calculated. When the studies reported both the crude and adjusted OR/RRs, the adjusted OR/RRs were extracted. Data extraction was carried out by two authors (Guo Zhi and Xing Guohong).

### Statistical Analysis

This meta-analysis of included studies was carried out with RevMan5.2and Stata 10.0. We abstracted odds ratios (OR), risk ratios(RR), and 95% confidence intervals (CI) from all studies. We then calculated logarithm of the OR (logOR) and its standard error (SE[logOR]) for all studies. The data were analyzed to generate a pooled effect size and 95% CI. A p value ≤ 0.05 was considered statistically significant. The heterogeneity of included studies was examined by the I^2^statistic. The summary estimates were obtained by the random effects model (P≤0.1, I^2^≥50% with heterogeneity) or the fixed effects model (P>0.1, I^2^<50% with no heterogeneity)[15].Subgroup analysis was used to assess heterogeneity between studies. We evaluated the possibility of publication bias with Egger’stest (Stata 10.0)[16].

## Results

### Literature Search and Study Characteristics

**Fig 1:**
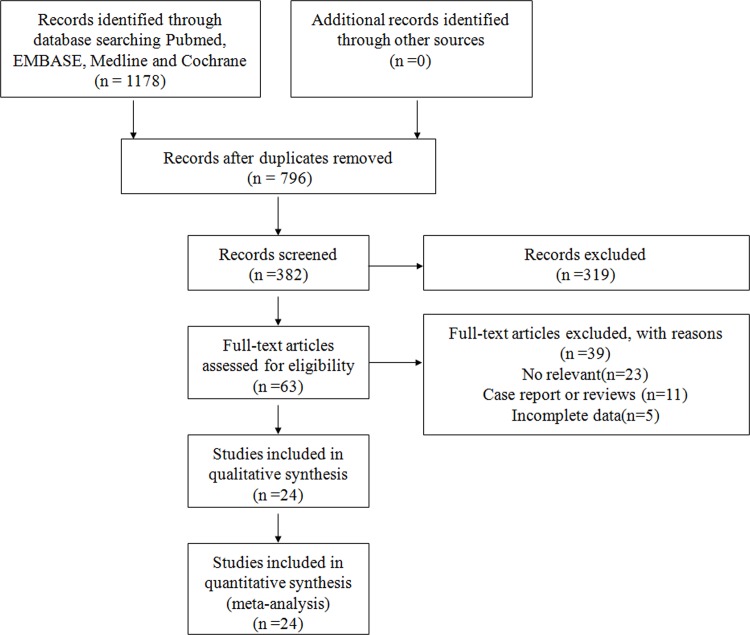
Study flow for the meta-analysis.

The method used to select studies is shown in Fig 1. 1178 articles were initially identified. After exclusion of duplicates(n = 796) and irrelevant studies (n = 319), 63 potentially eligible studies were selected.39 articles were excluded since they were case report or reviews (n = 10), did not evaluate obesity on ARDS/ALI (n = 20), or haven't provided sufficient data to further calculate(n = 9). Consequently, we identified 24 studies [8–14,17–33] that met our inclusion criteria. The characteristics of included articles are shown in Table 1 and Table 2.

**Table 1:** Characteristics of included studiesthat examined the effect of obesityonmorbidity of ARDS/ALI.

Author	Year	Studydesign	Sample size	BMI classification(kg/m^2^)	ARDS/ALI diagnostic standard	Outcomes
Anna M. Bramley	2012	retrospective Study	154	normal (BMI<30), obese (BMI 30–39.9), morbid obese (BMI ≥40)	NA	Morbidity: OR 1.928, 95% CI 0.718–5.181
Binod Dhakal	2013	retrospective study	320	normal (BMI<25), obese (BMI ≥25)	NA	Morbidity: OR 0.3, 95% CI 0.027–3.349
Huang Lei	2013	retrospective study	184	NA	American European Consensus Conference definition*	Morbidity: OR 5.023, 95% CI 2.486–10.150
I Bonmarin	2015	retrospective study	3074	normal (BMI<30), obese (BMI ≥30)	NA	Morbidity: OR 1.8, 95% CI 1.1–3.0
Jessica A. Palakshappa	2016	prospective study	164	underweight (BMI<18.5), normal (BMI 18.5–25), overweight (BMI 25–30), obese (BMI ≥30)	ARDS: both PaO2/FiO2 ratio<200 and bilateral infiltrates on chest radiographs within a 24-hour period	Morbidity: OR 2.388, 95% CI 1.075–5.305
John C. Weinlein	2015	retrospective study	507	normal (BMI<25), overweight (BMI 25–29.9)obese (BMI 30–39.9), morbid obese (BMI ≥40)	ARDS: PaO2/FiO2 ratio<200 for >12 hours, bilateral infiltrates on chest radiographs, and no evidence of a cardiogenic cause.	Morbidity: OR 35.38, 95% CI 1.79–699.7;
Jonathan Elmer	2013	retrospective study	697	normal (BMI≤30), obese (BMI >30)	ALI: diffuse, bilateral infiltrates on chestradiograph; at least two consecutive arterialblood gassamples with a PaO_2_:FiO_2_ratio < 300 mmHg; no evidence of left atrial hypertension.	Morbidity: OR 1.67, 95% CI 1.25–2.24
Juan C. Duchesne	2009	retrospective study	12759	normal (BMI≤29), obese (BMI 30–39.9), morbid obese (BMI ≥40)	American European Consensus Conference definition	Morbidity: OR 1.59, 95% CI 0.90–2.82
Lesly A. Dossett	2008	prospective study	1291	National Heart, Lung, and Blood Institute guidelines#	ARDS: the presence of bilateral patchy infiltrates seen on a chest radiograph; a PaO_2_/fraction of inspired oxygen ratio of < 200; and the absence of cardiogenic pulmonary edema	Morbidity: OR 0.36, 95% CI 0.13–0.99
Lioudmila V. Karnatovskaia	2014	prospective study	5584	National Heart, Lung, and Blood Institute guidelines	American European Consensus Conference definition	Morbidity: OR 1.69, 95% CI 0.98–2.8
Mark A Newell	2007	retrospective study	1543	normal (BMI 18.5–24.9), overweight (BMI 25–29.9), obese (BMI 30–39.9), morbid obese (BMI ≥40)	NA	Morbidity: OR 3.675, 95% CI 1.237–10.916
Michelle Ng Gong	2010	prospective study	1795	National Heart, Lung, and Blood Institute guidelines	American European Consensus Conference definition	Morbidity: OR 1.78, 95% CI 1.12–2.92;
Monisha A. Kumar	2012	retrospective study	69	normal (BMI<30), obese (BMI ≥30)	ARDS: PaO2/FiO2 ratio <200, bilateral lung infiltrates by radiography and a central venous pressure <18 mmHg. ALI: a PaO2/FiO2 ratio <300.	Morbidity: OR 2.01, 95% CI 0.489–8.27
Natalia Hagau	2010	prospective study	32	normal (BMI≤30), obese (BMI >30)	American European Consensus Conference definition	Morbidity: OR 13.33, 95% CI 1.434–123.98;
Shihua Yao	2013	prospective study	364	normal (BMI≤28), obese (BMI >28)	American European Consensus Conference definition	Morbidity: OR 3.25, 95% CI 0.392–26.916
Shirin Towfigh	2009	prospective study	2046	normal (BMI<30), obese (BMI≥30)	American European Consensus Conference definition	Morbidity: OR 1.57, 95% CI 1.01–2.45

BMI, body mass index; ALI, acute lung injury; ARDS, acute respiratory distress syndrome.

*American European Consensus Conference definition:the presence of bilateral patchy infiltrates seen on a chest radiograph; hypoxemia (PaO_2_ /FiO_2_< 300-ALI, PaO_2_ /FiO_2_< 200-ARDS); the absence of cardiogenic pulmonary edema.

#BMI classification by National Heart, Lung, and Blood Institute guidelines: underweight (BMI≤18.5), normal (BMI 18.5–25), overweight (BMI 25–29.9), obese (BMI 30–39.9), morbid obese (BMI ≥40)

**Table 2:** Characteristics of included studiesthat examined the effect of obesityonmortality of ARDS/ALI.

Author	Year	Studydesign	Sample size	BMI classification(kg/m^2^)	ARDS/ALI diagnostic standard	Outcomes
Amy E. Morris	2007	prospective study	825	National Heart, Lung, and Blood Institute guidelines#	American European Consensus Conference definition*	Mortality: OR 0.513, 95% CI 0.268–0.984;
Audrey De Jong	2013	retrospective Study	66	normal (BMI<30), obese (BMI≥35)	American European Consensus Conference definition	Mortality: OR 0.266, 95% CI 0.087–0.816
Ayman O. Soubani	2015	retrospective Study	2914	National Heart, Lung, and Blood Institute guidelines	NA	Mortality: OR 1.018, 95% CI 0.671–1.545
Graciela J Soto	2012	retrospective study	751	National Heart, Lung, and Blood Institute guidelines	American European Consensus Conference definition	Mortality: OR 0.81, 95% CI 0.71–0.93
James M. O’Brien Jr	2004	retrospective study	902	normal (BMI 18.5–24.9), overweight (BMI 25–29.9), obese (BMI 30–39.9), morbid obese (BMI ≥40)	NA	Mortality: OR 1.111, 95% CI 0.693–1.782
James M. O’Brien Jr	2006	retrospective study	1488	normal (BMI 18.5–24.9), overweight (BMI 25–29.9), obese (BMI 30–39.9), morbid obese (BMI ≥40)	NA	Mortality: OR 0.78, 95% CI 0.44–1.38
Michelle Ng Gong	2010	prospective study	1795	National Heart, Lung, and Blood Institute guidelines	American European Consensus Conference definition	Mortality: OR 0.89, 95% CI 0.71–1.12
Renee D. Stapleton	2010	retrospective study	1409	National Heart, Lung, and Blood Institute guidelines	American European Consensus Conference definition	Mortality: OR 0.425, 95% CI 0.227–0.794
Stavros G. Memtsoudis	2011	retrospective study	9149030	NA	NA	Mortality: OR 0.31, 95% CI 0.28–0.36

BMI, body mass index; ALI, acute lung injury; ARDS, acute respiratory distress syndrome.

*American European Consensus Conference definition: the presence of bilateral patchy infiltrates seen on a chest radiograph; hypoxemia (PaO_2_ /FiO_2_< 300-ALI, PaO_2_ /FiO_2_< 200-ARDS); the absence of cardiogenic pulmonary edema.

#BMI classification by National Heart, Lung, and Blood Institute guidelines: underweight (BMI≤18.5), normal (BMI 18.5–25), overweight (BMI 25–29.9), obese (BMI 30–39.9), morbid obese (BMI ≥40)

### Meta-Analysis Results

#### Obesity and morbidity of ARDS/ALI

**Fig 2:**
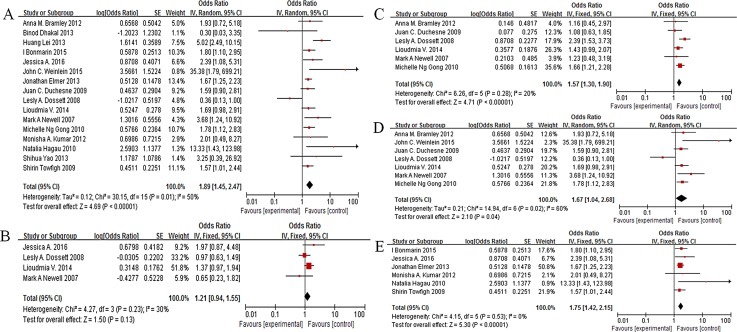
Association of obesity and morbidity in ARDS/ALI patients. A: pooled result; B: overweight(BMI25-29.9kg/m^2^) vs normal; C: obese(BMI30-39.9kg/m^2^) vs normal; D: morbid obese(BMI≥40kg/m^2^) vs normal; E:obese(BMI≥30kg/m^2^) vs normal.

As shown in Fig 2A, Obesity was associated with a significantly increased risk of ARDS/ALI(pooled OR = 1.89, 95% CI: 1.45–2.47, I^2^ = 50%, P<0.00001, n = 30583,Fig 2A).3 included studies (n = 8670) reported that patients were classified into the following BMI groups according to the National Heart, Lung, and Blood Institute guidelines[34]: underweight (BMI≤18.5m^2^), normal (BMI 18.5-25m^2^), overweight (BMI 25–29.9m^2^), obese (BMI 30–39.9m^2^), morbid obese (BMI ≥40m^2^). In the subgroup analysis by this classification, compared to the normal weight, overweight (BMI 25-30kg/m^2^)was not associated with risk of ARDS/ALI(OR = 1.21, 95% CI: 0.94–1.55, P = 0.13, I^2^ = 30%, n = 6540,Fig 2B).Six studies evaluated obese (BMI 30–39.9kg/m^2^) inrisk of ARDS/ALI, and OR was 1.57(OR1.57, 95% CI 1.30–1.90, P<0.00001, I^2^ = 20%, n = 5426, Fig 2C). For morbid obese (BMI ≥40kg/m^2^), there was a 67% increase in risk of ARDS/ALI (OR1.67, 95% CI 1.04–2.68, P = 0.04, I^2^ = 60%, n = 4124, Fig 2D).On the other hand, 6 studies defined obesity as a BMI≥30kg/m^2^. Obesity(BMI≥30kg/m^2^) was associated with a significantly increased risk of ARDS/ALI (OR1.75, 95% CI 1.42–2.15, P<0.00001, I^2^ = 0%, n = 6025, Fig 2E).

#### Obesity and mortality of ARDS/ALI

**Fig 3:**
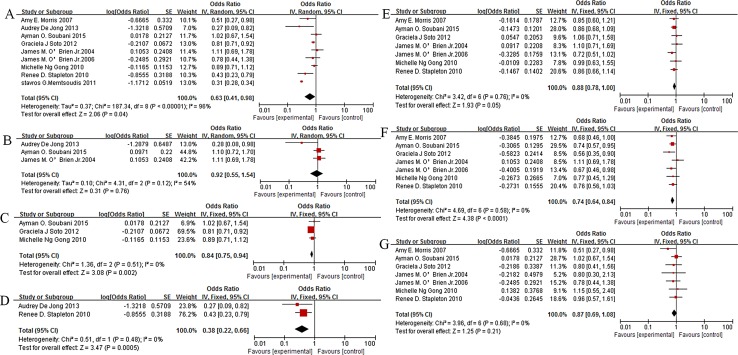
Association of obesity and mortality in ARDS/ALI patients. A:pooled result; B: 28days; C: 60days; D: 90days;E: overweight vs normal; F: obese vs normal; G: morbid obese vs normal.

Fig 3 showed the association between obesity and mortality of ARDS/ALI. Obesity was associated with lower risk of mortality in ARDS/ALI (pooled OR 0.63, 95% CI 0.41 to 0.98,P = 0.04,I^2^ = 96%,n = 9159180, Fig 3A).To clarify the heterogeneity, subgroup analysis was stratified according to BMI classification[34]and mortality in different periods. Three studies were plotted and showed no obvious relationship between obesity and 28days mortality in ARDS/ALI (OR0.92, 95% CI 0.55–1.54, P = 0.76, I^2^ = 54%, n = 1723, Fig 3B).Obesity was associated with lower risk of 60 days and 90 days mortality in ARDS/ALI(60days: OR0.84, 95% CI 0.75–0.94, P = 0.002, I^2^ = 0%,n = 1946, Fig 3C;90days:OR0.38, 95% CI 0.22–0.66, P = 0.0005, I^2^ = 0%,n = 682, Fig 3D). In reference to the normal weight, overweight and obese were associated with decreased mortality risk (overweight:OR0.88, 95% CI 0.78–1.00,P = 0.05, I^2^ = 0%, n = 6155, Fig 3E; obese: OR0.74, 95% CI 0.64–0.84,P<0.0001, I^2^ = 0%,n = 5488, Fig 3F).However, there was no evidence of a relationship between morbid obese and ARDS/ALI mortality (OR0.87, 95% CI 0.69–1.08,P = 0.21, I^2^ = 0%,n = 4145, Fig 3G).

### Publication Bias

We detected no publication bias among the included studies that assessed obesity in morbidity and mortality of ARDS/ALI based on Egger’s test (P = 0.331,S1 Fig;P = 0.530,S2 Fig).

## Discussion

This current meta-analysis, including 9,187,248 subjects from 24 observational studies, explored the association between obesity and risks of ARDS/ALI. The results of our meta-analysis indicated that ARDS/ALI morbidity was higher for patients with obesity compared to normal weight. In addition, obesity was significantly associated with a reduction in ARDS/ALI mortality, especially in long-term mortality.

In subgroup analyses by BMI classification, except for overweight, we found that obese and morbid obese could increase the risk of ARDS/ALI. Moreover, it was confirmed by our result in another obesity group (BMI≥30kg/m^2^).There were several potential explanations for why obesity may have higher risk of ARDS/ALI. First, obesity is a chronic inflammatory disease. Patients who are obese have increased circulating neutrophil levels[35,36] and elevations in blood cytokines such as TNF-α, IL-1β, IL-8 and IL-6[37,38].Some studies in mice have shown that increased inflammatory cytokines induce depletion of anti-oxidant stores, up-regulation of adhesion molecules on lung endothelium and enhanced susceptibility of endothelium to injury [39,40]. Moreover, innate immune cell activation and endothelial injury in the pulmonary microvasculature is a major contributor to the increased permeability pulmonary edema of ARDS/ALI in obese patients[41–43].Another significant feature of obesity is the dysregulation of adipokine release and response. Shah et al[44] found that Obesity-induced adipokine imbalance impaired pulmonary vascular homeostasis, altered the expression of endothelial junctional adherens and adhesion proteins, and primed the lung for acute injury. Moreover, these hormonal changes, inflammation-related actions and immune-related activities have been shown to be important in the development of ARDS/ALI [45–47]. Finally, obese patients experience a number of changes in pulmonary mechanics compared to lean individuals. Some of the changes in obese patients include a decrease in total lung capacity (TLC), functional residual capacity (FRC), and vital capacity (VC) as well as consequently atelectasis, increased airways resistance and closure, and ventilation/perfusion mismatch[48,49].These findings strongly support a link between obesity and risk of developing ARDS/ALI.

Our meta-analysis suggested a survival advantage for obese patients with ARDS/ALI and supported the emerging concept of the ‘‘obesity paradox.”

The mechanism of the ‘‘obesity paradox” in patients with ARDS/ALI was unclear. We propose some explanations for the inverse relationship between obesity and the risk of ARDS/ALI mortality. First, a recent study analyzed plasma biomarker levels in 1409 patients with ARDS and found that obese patients have lower levels of proinflammatory cytokines (IL-6, IL-8) and surfactant protein D (a marker of alveolar epithelial injury) compared to normal weight patients[30].Moreover, recent animal studies[50,51] suggested that obesity-associated attenuation of ALI was in part attributable to an obesity-associated abnormal neutrophil chemoattractant response. These studies indicated that obesity may be “primed” for the development of ARDS/ALI, but innate immunity and the inflammatory response may be altered and attenuated during ARDS/ALI. Second, obese patients may receive optimal medical treatment or aggressive treatment for an increase risk of cardiovascular disease and diabetes [52].Finally, obese patients may provide a metabolic reserve to counteract the increased catabolic stress of ARDS/ALI because of additional energy stores in the form of adipose tissue. Additional energy stores and optimal medical treatments in the obese patients may explain the improved long-term mortality of ARDS/ALI in our results. Interestingly, consistent with a U-shaped relationship between BMI and mortality[53–55],we found obesity(BMI30-39.9kg/m^2^) had the lowest ARDS/ALI mortality and no obvious relationship between morbid obese(BMI≥40kg/m^2^)and ARDS/ALI mortality.

Our study has some potential limitations. First, since this is an observational meta-analysis, it inherits the limitation of the original studies. The effect of confounding, such as age, gender, and underlying diseases, was not examined. For example, pre-existing diabetes was associated with a decreased risk of ALI/ARDS [56]. In this meta-analysis, 8 studies included diabetes patients and 8 studies did not report it clearly. For insufficient data, we did not assess the effect of diabetes on the development of ARDS/ALI in patients with obesity. Second, obesity was defined by different methods in different studies, such as BMI >28kg/m^2^ and BMI >25kg/m^2^, which were excluded in subgroup analysis. Third, high heterogeneity was found in subgroup, especially for obese patients in 28days mortality and morbid obese patients in risk of ARDS/ALI. Given this limitation, the results should be carefully interpreted, and confirmed by future studies.

In conclusion, obesity is associated with an increased risk of ARDS/ALI, but obese patients have a lower mortality risk when compared to patients with a normal BMI. Our results support the emerging concept of the ‘‘obesity paradox” in ARDS/ALI.

## Supporting Information

S1 Checklist The PRISMA checklist. (DOC)

S1 Fig Egger’s test in morbidity of ARDS/ALI. (TIF)

S2 Fig Egger’s test in mortality of ARDS/ALI. (TIF)
