# Supplementary figures and images for: “Obesity Paradox” in Acute Respiratory Distress Syndrome: Asystematic Review and Meta-Analysis
Source: PLoS One. 2016 Sep 29;11(9):e0163677. doi: 10.1371/journal.pone.0163677 (PMC5042414; doi:10.1371/journal.pone.0163677)

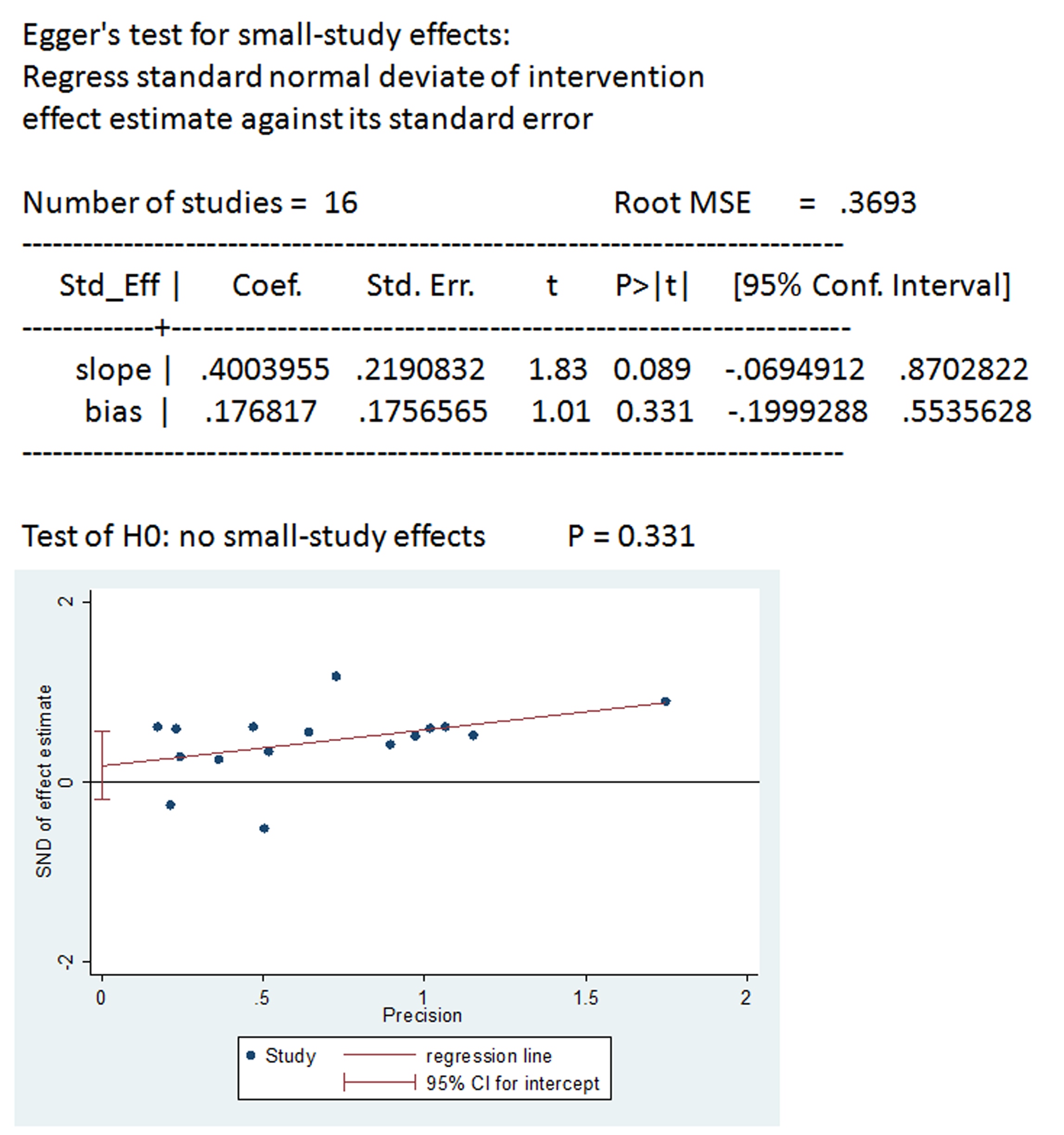

Supplement: S1 Fig — (TIF) [file pone.0163677.s002.tif]

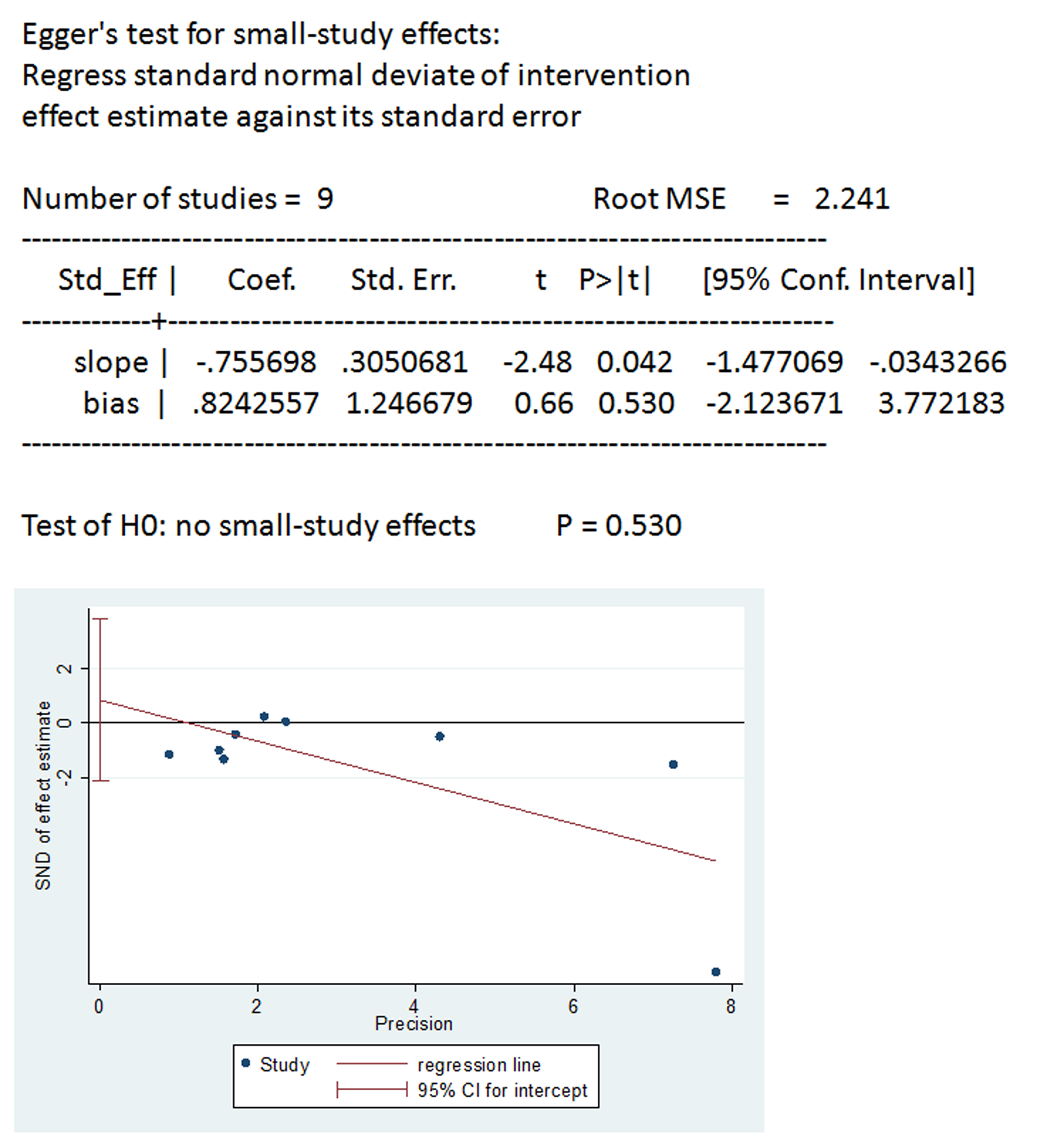

Supplement: S2 Fig — (TIF) [file pone.0163677.s003.tif]
